# How Do Older Adults in a Sub-Saharan African Community Perceive and Cope With Their Disability? An Interpretive Phenomenological Analysis

**DOI:** 10.3389/ijph.2023.1606273

**Published:** 2023-11-23

**Authors:** Judith Ifunanya Ani, Kezia Batisai, Loretta F. C. Ntoimo, Uche C. Isiugo-Abanihe

**Affiliations:** ^1^Department of Sociology, Faculty of Humanities, University of Johannesburg, Johannesburg, South Africa; ^2^ Department of Demography and Social Statistics, Faculty of Social Sciences, Federal University Oye-Ekiti, Oye, Nigeria; ^3^ Department of Sociology, Faculty of the Social Sciences, University of Ibadan, Ibadan, Nigeria

**Keywords:** older adults, disability, coping strategies, interpretive phenomenological analysis, Sub-Saharan Africa

## Abstract

**Objective:** The study explored the perceptions and coping strategies employed by older adults in a Sub-Saharan African community in relation to their disabilities.

**Methods:** The research utilized an Interpretive Phenomenological Analysis methodology and conducted semi-structured interviews with a purposive sample of households. The study recruited a total of 36 older adults aged 65 years and above, aiming to capture a diverse range of insights and perspectives within the Sub-Saharan African community.

**Results:** Three interrelated themes pertaining to the perception of disability emerged: the impact of old age, disabilities caused by diseases, and disabilities attributed to external factors such as witchcraft. In coping with disability, two interrelated themes emerged: pragmatic coping strategies and unpragmatic coping strategies.

**Conclusion:** This study offers valuable insights into the nuanced perception of disability and coping mechanisms utilized by older adults within the Sub-Saharan African community. By exploring their lived experiences, the findings contribute to a better understanding of the challenges they face. These insights have important implications for policy development and public health initiatives.

## Introduction

Sub-Saharan Africa is projected to experience significant population growth in the coming decades, including a rise in the number of older adults. The population of older adults aged 65 years and above in the region is expected to increase from 32 million in 2019 to 101 million by 2050 [1, 2]. Consequently, Scholars emphasize the need to strengthen care and support systems for older adults in this region to address the impending challenges [3, 4]. Research, policies, and programs are also called for to improve the wellbeing of older adults and mitigate potential negative impacts on public health [5, 6]. Ageing correlates with higher disability rates, diminishing independence [7]. It also associates with anxiety, restrictions, health challenges, and difficulties [8]. In developing nations with high poverty rates, ageing and disability are often neglected policy concerns [9]. Disability is defined as limitations from impairments, hindering daily activities and functional independence [10]. Disability is intricate, requiring coping strategies involving cognitive and behavioral approaches to manage challenges [11]. The perception of disability, akin to coping, significantly influences health outcomes [12]. Recognizing and addressing the complexity of disability, while promoting effective coping, can enhance wellbeing and overall health for individuals with disabilities.

Coping is categorized into types like “active versus passive,” “approach versus avoidance,” “problem-focused versus emotion-focused,” and “adaptive versus maladaptive” [13, 14]. Active, approach, problem-focused, and adaptive coping maintain control and address stressors, while passive, avoidance, emotion-focused, and maladaptive coping involve resignation and inefficient defense mechanisms. Coping also involves engagement (focusing on the problem, seeking support) and disengagement (employing defense mechanisms, denial) [15]. In engagement coping, individuals find meaning through values and beliefs, while disengagement coping, in uncontrollable situations, may involve reliance on religious beliefs and denial.

Considering the interconnectedness of individuals in social structures [16], exploring disability perception and coping through Interpretive Phenomenological Analysis (IPA) is crucial. IPA unveils themes in personal narratives [17], offering insight into overlooked aspects in lived experiences. It links individual experiences with broader societal and global interpretations, providing a comprehensive understanding of how individuals navigate disability challenges in their social and cultural contexts. Understanding how older adults perceive disability and cope is crucial for policy development and public health improvement. Research exploring their perspectives, challenges, and coping mechanisms can inform tailored support systems and interventions to improve wellbeing and quality of life. This study, therefore, explores disability perceptions and coping strategies among older adults in a Sub-Saharan African community, providing an interpretive perspective on the dual challenges of old age and disability, contributing to gerontological literature, and revealing the interplay between individual and societal contexts.

## Methods

### Design

This qualitative study employed in-depth interviews and the Interpretive Phenomenological Analysis (IPA) framework to explore how older adults perceive and cope with disabilities. Following IPA’s recommended stages [18], from formulating questions to data analysis, the research aimed for a hermeneutic interpretation of participants’ experiences. The rigorous approach provided deep insights into their perspectives and coping mechanisms, capturing nuanced aspects of older adults’ experiences with disabilities.

### Study Location and Participant Sample

The study addressed the dearth of data on older adults with disabilities and their coping strategies in an Igbo-speaking community in Enugu State, south-eastern Nigeria. Purposive sampling involved recruiting participants aged 65 or older, living with a disability for at least 12 months. Community gatekeepers identified eligible participants, and disability determination relied on self-reported need for assistance in at least one Activity of Daily Living (ADL) or Instrumental Activity of Daily Living (IADL). Communication was facilitated by two trained research assistants proficient in participants’ dialects. The study included 36 participants, meeting criteria for theoretical saturation [19] and following the Interpretive Phenomenological Analysis (IPA) methodology [20].

### Interviews

The study employed a semi-structured interview approach to gather data on participants’ demographic characteristics, perceptions of disability, coping strategies, societal dynamics, governmental involvement, and challenges of living with disability in old age. Researchers, skilled in qualitative research and Interpretive Phenomenological Analysis (IPA), conducted interviews. In cases where coherent responses were not possible, interviews were discontinued for accurate reflection. The hour-long interviews, conducted in participants’ homes for comfort, were audio-recorded in both English and Igbo. Igbo interviews were translated and transcribed for analysis and interpretation.

### Research Quality

The study adhered to key principles such as relevance, context sensitivity, coherence, transparency, rigour, and commitment to the research process. Emphasizing relevance, the study aimed to inform policy and public health initiatives, particularly focusing on older adults living with a disability. Context sensitivity was evident in aligning with research objectives. Transparency was maintained through a detailed description of participant recruitment and data analysis, enhancing study credibility. Rigour and commitment were upheld by researchers through attentive interviews and thorough transcriptions analysis. They acknowledged personal experiences but demonstrated willingness to set aside biases, displaying expertise in using the Interpretive Phenomenological Analysis (IPA) methodology, reaffirming their dedication to the research.

### Analysis

Transcript analysis followed a methodical approach, involving reading for familiarity, identifying significant statements, coding, and grouping into themes. Manifest and latent content were considered, aiming to reveal both surface-level and deeper meanings. Integrity, trustworthiness, and credibility were prioritized for a balanced representation. Reflexivity acknowledged biases, and researchers consciously bracketed perspectives [21–24] for objectivity. Pseudonyms protected identities, and coping strategies were categorized as “pragmatic” or “unpragmatic.” The study adhered to rigorous procedures, ensuring an accurate and nuanced interpretation while maintaining transparency and credibility.

### Ethics Approval and Consent to Participate

This study was approved by the University of Ibadan Social Sciences and Health Research Ethics Committee (UI/SSHREC/2019/0006). The study was conducted in accordance with the local legislation and institutional requirements. The participants provided their written consent to participate in this study. Ethical considerations such as respect for persons, confidentiality, voluntariness, non-malfeasance, and sensitivity were carefully taken into account throughout the study.

## Results

### Participants Information

The study included 36 participants, with a higher female representation (58.3%). The majority (47.2%) were aged 65–69, and nearly 70% were married, with over 80% having more than four children. Only 5.6% had tertiary education, and none were employed. The prevalent disabilities reported were mobility-related (e.g., from stroke) and pain-related. (See Table 1).

**TABLE 1:** Demographic characteristics of older adults with disabilities (Enugu, 2021).

Variable	Frequency (*N* = 36)	Percentage (100%)
Gender		
Male	15	41.7
Female	21	58.3
Age		
65–69	17	47.2
70–74	9	25.0
75–79	6	16.7
80+	4	11.1
Mean Age: 71.75		
Marital Status		
Currently Married	25	69.4
Previously Married	11	30.6
Number of Children Had		
None	2	5.6
1	0	0.0
2	0	0.0
3	5	13.9
4 and above	29	80.6
Highest Level of Education		
No Formal Education	5	13.9
Primary	17	47.2
Secondary	12	33.3
Tertiary	2	5.6
Currently in the Labour Force		
Yes	0	0.0
No	36	100
Type of Disability Participants Had		
Mobility-related (Stroke)	15	41.7
Sight-related (Blindness)	5	13.9
Pain-related (rheumatoid arthritis)	14	38.9
Dementia	2	5.6

### Perception of Disability

Participants’ disability perceptions were categorized into three themes: the effect of old age, attributing disability to diseases, and perceiving disability as inflicted by external forces like witchcraft. The first theme sees disability as a natural consequence of aging. The second emphasizes the belief that diseases or health conditions caused disabilities. The third theme reflects a view where some participants see their disabilities as inflicted by external factors, indicating a more adversarial perspective.

Specifically on the effect of old age, participants said:

…I am old and my body is weak. I am not able to do things I used to because I am old. It is very difficult to move around because of the pain… -Paul

…When one begins to grow old, he will not be able to do things they were used to in their younger days… -Obigwe

…Old age is beautiful but it comes with some problems. You wake up one morning and find out things are changing. You are not able to wake up as you used to before… -Sussana

I used to encourage younger ones to take care of their health in their youth because old age has a lot of challenges it gives to people who are not prepared. -Theresa

On their disabilities being caused by disease, participants expressed their perspectives in the following manner:

…My blindness was caused by a disease. That was what my doctor told me… -Nneoma

Sometimes, I feel my hands shake and I cannot see clearly. The doctor told me that the reason why my hands shake is called Parkinson disease… -Ejike

…Something happened to me when I was a child and my parents did not treat it medically. They were going to native doctors who gave them some herbs that I took. Before they could get me to the hospital after some months, I was already blind. The doctor said it was a disease that could have been treated but because they came in late, the damage was already so much… -Mazi Okeke

…My knees pain me so much. It is caused by what the doctor called rheumatoid arthritis. The pain is excruciating, especially in the morning when I wake up. I have been treating it. I take painkillers and other prescribed medications… -Mama Ifeanyi

Participants, in these accounts, recognized a close link between their disabilities and specific diseases or health conditions. They associated disability onset with disease progression, acknowledging the profound impact on physical functioning. By understanding this connection, participants emphasized the importance of managing and treating underlying health conditions to potentially alleviate or mitigate resulting impairments.

Participants described their disabilities as inflicted by an enemy, conveying a sense of intentional harm. Phrases like “My disability was like an attack” or “It felt like someone cast a spell on me” reflected a belief in malicious intent or deliberate harm from an external source. By framing their disabilities in this way, participants attributed a personal struggle and perceived themselves as victims of a hostile force.

Accordingly:

I do not doubt that my sickness was sent by my enemies through accident. I was on my way from the market, about to cross the road to the other side; a careless driver knocked me down and ran away. How do I explain this if not an evil attack? …-IgweAgu

Some people are not happy because you are progressing. What do you call them but witches? They are enemies. They see you happy and the next thing is that they send an arrow against you spiritually and then people will call it sickness but I know it is spiritual. How do you explain walking and suddenly, something enters your eyes and like a joke, you become blind? -Innocent

I was on my way from the market when I suddenly felt a sharp pain in my head. I screamed and that was the beginning of my trouble. We did some consultations with the herbalist and he said someone sent stroke sickness to me. I began noticing some changes in my body. He gave me some herbs that would help but unfortunately, it did not work because it is spiritual. That was how I became unable to stand or do anything. It was like a film, the type they do on Africa Magic Nollywood but here I am. People are evil. Witches can cast a spell and see me today, down with a stroke. Unbelievable! -Mama Chiegeonu

### Coping With Disability

Through our analysis, we identified two major coping strategies employed by older adults with disabilities. These coping strategies were categorized as pragmatic and unpragmatic coping strategies. By categorizing coping strategies as pragmatic and unpragmatic, we aimed to capture the diverse approaches that older adults with disabilities employ to navigate their daily lives.

### Pragmatic Coping

Pragmatic coping signifies acceptance and understanding of disability. Participants employ active, problem-focused approaches to manage disabilities, aiming to regain control, maintain independence, and make positive changes in their lives. These strategies reflect resilience and determination to overcome challenges, maintaining a sense of agency in managing disabilities.

For instance, a participant who sought medical attention to cope with his disability said:

I am already old. My body is weak. Living with a disability is difficult. To help me survive until God calls me, I try as much as possible to get medical attention always. The last time I was at the hospital for a check-up, the doctor told me my blood pressure was rising. He gave me some drugs and I feel better now. I wonder if I had not visited. I probably may have died of high blood pressure without knowing -Sam

Surviving daily, especially through remittance was one of the major hallmarks of adaptive (pragmatic) coping daily. To them, their ability to adapt to their disability was contingent on the support they received.

A participant said,

Not only do I have to cope with my disability daily, but I have to ensure that I survive daily too. While I commit my difficulty to God, I look up to my children for support. My children are my source of support. I do not have any other means. They provide for me by sending money for me to care for myself. -Patricia

### Unpragmatic Coping

Participants also discussed unpragmatic coping strategies, which were characterized by a more passive, state of helplessness and emotion-focused approach to dealing with their disabilities. These strategies involved resignation, denial, a sense of helplessness and abandonment by the government in the face of their impairments. Participants who adopted unpragmatic coping strategies often expressed feelings of sadness, frustration, and withdrawal.

Accordingly,

…My enemies have seen my destiny and they want to destroy it but they have failed. My pastor told me that it was an attack. Even a native doctor said the same thing. Some rituals were done for cleansing and we have also been praying since then. It is over 5 years now. I feel so helpless… -NwanyiOji

Participants emphasized societal failures, particularly by the government, during interviews. They expressed frustrations and disappointment with inadequate support for older adults with disabilities, citing a lack of infrastructure, limited healthcare services, and insufficient social support systems. Participants felt overlooked, limiting their ability to cope with disabilities.

Specifically,

…It is not easy having to depend on someone for your livelihood but what do we do since we are out of the government coverage. There are no provisions for us… We lack government support and it makes it more difficult to cope every day. The government has failed us. Our society has failed us. …Assessing healthcare is difficult… I wish the government can look towards us to help us. It is not the fault of any old person to be in this condition. It is frustrating… -Mama Okechukwu

I have to beg daily for my sustenance. Begging is not a pleasant experience. Some will look down on me thinking that I am a ritualist who wants to use their money for diabolic purposes. Some will just throw twenty naira (20.00) at me, although it is better than nothing. Some days the rain will beat me and I get cold. On other days, the sun will make me regret my life. There are some days I do not get up to one hundred naira (100.00). It is not easy at all. I wish the government could do something for us at least for once so we do not die before our time -Mama Ijeoma

Another who also begged for alms said:

… The government has not helped in any way. I go out to beg by the roadside. People help me with money and that is how I survive… It is not easy at all. Sometimes, you can stay by the roadside all day to beg for alms and receive only two hundred naira (200.00) in a whole day. It is so sad…-Roseline

Reflecting further, another accounted:

I was born in 1946… My husband is late… As you can see, I can’t walk and I have been living like this for about 45 years… I suffered so much in my life but I thank God. I am so weak now. It is so tough. If not for the church and private individuals, I do not know what would have become of me. When I do not receive alms from them, I struggle to get to the roadside to beg passers-by for alms. Some of them who are touched by my condition drop money for me… I beg for food and money. Sometimes when it rains, I get drenched because I sit under a thatched roof to beg… I am dying of hunger… Begging is not a pleasant experience. The government has made life miserable for us… -Mama Nkechi

Acknowledging societal failures underscores the importance of comprehensive support systems and society’s proactive role in promoting the wellbeing and rights of older adults with disabilities. It highlights the need for policy changes and collective efforts to address their needs, ensuring dignity, care, and support rather than allowing them to be left in helplessness and frustration due to their disabilities.

In our analysis, we observed a coping mechanism of alms begging, not just due to a lack of formal government assistance but also influenced by the economic hardships faced by their children. Participants shared relying on alms begging to meet basic needs and financial support, as their children could not adequately provide. Older adults with disabilities resorted to seeking assistance through begging due to insufficient financial support from their children.

…I beg for alms not because I do not have children but because they do not have good jobs and cannot support me adequately… -Nweze

My children find it difficult to provide for themselves. It is even more burdensome for them to provide for me. The economy is hard. Everything is expensive even food. It is so sad and I am not happy that my children cannot cater comfortably for themselves and talk more of me…-Nnabuike

These accounts reveal the intricate interplay between economic factors, family dynamics, and coping strategies. Narratives underscore the financial strain on older adults with disabilities and their families, influencing coping mechanisms like alms begging. The interconnectedness of social and economic factors in shaping their experiences is evident. Findings stress the need for comprehensive social welfare programs, economic support, and employment opportunities to alleviate financial burdens and offer alternative support for older adults with disabilities. Addressing economic hardships can promote more sustainable and dignified coping strategies, reducing reliance on alms begging for survival.

Dependence on remittances has emotional and psychological implications for older adults with disabilities. Participants relied on financial support from family members in other locations, often through remittances, to cope with disabilities and meet basic needs. However, this dependence brought feelings of insecurity, vulnerability, and a sense of burden on supporting family members. The uncertainty of remittances, which could fluctuate or cease, led to anxiety and emotional distress among participants.

…Although my children do their best to provide for me it is not always easy to depend entirely on them as it affects my emotions and I become worried when I do not hear from them or receive anything from them… -Mama Uka

Furthermore, participants described a loss of autonomy and independence as they had to depend on others for their financial wellbeing. This was seen as a reminder of their disability and a constant source of emotional strain. It also contributed to a sense of powerlessness and diminished self-esteem, as they felt unable to contribute to their financial support or make independent decisions regarding their lives.

…Depending on anyone has never been my style before now, I work hard. If not for my disability, I would not be waiting for my children to send anything before I take care of myself or eat. It is sad. My independence is gone. It is a whole new phase of life but there is nothing I can do about it… -Mazi Obinna

These findings highlight the complex emotional dynamics that arise from financial dependence for older adults with disabilities. While financial support is essential for their survival, it also poses emotional challenges and psychological implications, further leveraging a sense of helplessness in the face of their disability. Understanding these emotional and psychological dimensions is, therefore, crucial for developing support systems and interventions that address not only the financial needs but also the emotional wellbeing of older adults with disabilities.

## Discussion

The study explored perceptions and coping strategies of older adults with disabilities in a sub-Saharan African community. Notably, the community places high cultural value on children, evident in the participants’ substantial number of offspring. Mobility-related disabilities, mainly stroke, emerged as prevalent, with participants acknowledging stroke as a major cause of disability and mortality risk [25, 26].

In African cultures, disability perception extends beyond medical explanations, shaped by cultural and spiritual influences. In the studied community, older adults attribute disabilities to old age, disease, or affliction by an enemy. Old age is culturally esteemed, linked to wisdom, respect, and spiritual significance in this particular community [27]. However, it is also commonly believed that old age brings about physical and mental decline [28]. The understanding that disabilities are linked to old age arises from acknowledging the natural weakening of the body and increased vulnerability to health conditions with age. Consequently, disabilities are viewed as an expected outcome of the aging process.

Disease, notably stroke, is a prominent factor identified by older adults as a cause of disabilities in the community. Sub-Saharan Africa faces health challenges, including infectious diseases and limited access to quality healthcare. Chronic non-communicable diseases, like cardiovascular diseases, stroke, rheumatoid arthritis, diabetes, and respiratory conditions, pose a substantial health burden, especially among older adults. These chronic diseases have long-term implications for health and functioning [29–31]. The link between disabilities and diseases in older adults mirrors the health landscape and challenges in Africa, where infectious diseases and chronic conditions contribute significantly to disabilities. Recognizing prevalent diseases and their impact is crucial for implementing effective prevention, treatment, and management strategies. This emphasizes the need for public health interventions, improved healthcare access, and initiatives addressing the burden of infectious diseases and chronic conditions to reduce disabilities and enhance overall wellbeing in African communities.

In many African cultures, the belief in supernatural causation of disabilities, observed in the studied community, is deeply ingrained. Witchcraft, sorcery, and evil spirits are considered potential causes [32, 33], reflecting a worldview where disabilities result from malevolent intentions or spiritual attacks. This belief system serves to make sense of disabilities within cultural and spiritual contexts. These cultural beliefs significantly impact individuals with disabilities, influencing social interactions, stigma, and the availability and utilization of support services in African societies.

Coping with a disability, as observed in the study, involves two main approaches: pragmatic coping and non-pragmatic coping. Pragmatic coping strategies include seeking medical attention, enabling individuals to adapt, develop new perspectives, prioritize healthcare, and adopt a positive mindset to handle stressors. Older adults using pragmatic coping often show better social capital, fewer depressive symptoms, improved quality of life, and positive psychological wellbeing [34]. Recognition and acceptance of the inevitability of old age motivate them to seek healthcare to maximize their remaining time.

Perceiving disability as an affliction caused by an enemy may lead individuals to consult spiritualists for coping, aligning with personal beliefs. While religious coping can be positive [35, 36], participants in this study described a form of coping that leaned towards unpragmatic and negative aspects. This coping approach resulted in emotional distress, unmet recovery expectations, and adaptation difficulties. The findings align with the transactional theory of stress and coping [37], emphasizing the influence of perception, coping ability, and available resources on adaptive responses. Understanding individuals’ coping strategies and beliefs is crucial for developing interventions that promote effective coping and overall wellbeing.

Coping with disability in this community is significantly influenced by dependence on children, rooted in the cultural and economic importance placed on high fertility and intergenerational wealth flow. Scholars [38] argue that high fertility rates are rational in societies valuing both spiritual and economic aspects of marital fertility. This belief persists, with family structure and wealth flow linked, emphasizing the benefits of having many children for upward wealth flow to parents. Larger households are maintained, and the power of the household head is often tied to the number of children. Children are seen as an economic investment, relied upon for support in parents’ old age.

Contemporary Africa experiences a decline in support for the elderly due to limited resources among the younger generation [4]. This trend exposes older adults to poverty, alms begging, and destitution. The study, supported by previous findings [39], highlights government neglect of older adults’ welfare, echoing participants’ frustrations. Government inaction may stem from negligence, insufficient analysis of challenges, or an assumption that issues will self-resolve. Societal failure to address older adults’ welfare emphasizes the urgent need for government intervention and formal social welfare programs. Recognizing vulnerabilities and challenges is crucial for promoting wellbeing and addressing issues like poverty and destitution.

In this community, alms begging is prevalent, especially among older adults whose children lack the financial means to adequately support them. The absence of comprehensive aging programs in national development plans [40] contributes to poverty, leading to reliance on charity. Financial constraints faced by the younger generation limit their support for older adults [41]. The lack of a social safety net or pension scheme further compounds financial difficulties for elderly individuals not covered by such programs [42]. Many older adults in this community, having worked in the informal sector, lack savings or retirement funds, compelling some to resort to alms begging. Importantly, soliciting alms does not imply childlessness; scholars [43] argue it often results from children’s inability to provide, exacerbated by the absence of formal support structures in society.

### Conclusion and Recommendations

The study provides comprehensive insights into the perceptions and coping strategies of older adults with disabilities in a sub-Saharan African community, emphasizing cultural and spiritual influences. It identifies causes such as old age, diseases, and perceived affliction by enemies, and distinguishes pragmatic and unpragmatic coping approaches, highlighting the significant role of children for support. Challenges arise from declining familial support and limited social welfare systems, leading some to resort to alms begging. Recommendations include culturally sensitive interventions, prioritizing welfare policies, and addressing specific health conditions. The study urges government commitment through national development plans and social safety nets. Despite insights, limitations are acknowledged, and future research should employ mixed-methods approaches for a more nuanced exploration of older adults’ experiences with disabilities.

## References

[1] United Nations, Department of Economic and Social Affairs, Population Division. World Population Prospects: 2019 (ST/ESA/SER.A/423). United Nations: United Nations, Department of Economic and Social Affairs, Population Division (2019).

[2] United Nations, Department of Economic and Social Affairs, Population Division. World Population Ageing 2019: Highlights (ST/ESA/SER.A/430). United Nations: United Nations, Department of Economic and Social Affairs, Population Division (2019).

[3] Adamek M Chane S Kotecho M. Family and Kin Care of Elders in Sub-Saharan Africa. In: Maharaj P Mokomane Z, editors. Health & Care in Old Age in Africa. London: Routledge (2020). p. 61–77. 10.4324/9780429020759

[4] Ani IJ. Care and Support for the Elderly in Nigeria: A Review. Niger J Sociol Anthropol (2014) 12(1):9–27. 10.36108/NJSA/4102/12(0110)

[5] Adamek ME Gebremariam Kotecho M Chane S Gebeyaw G. Challenges and Assets of Older Adults in Sub-Saharan Africa: Perspectives of Gerontology Scholars. J Aging Soc Pol (2021) 34(1):108–26. 10.1080/08959420.2021.1927614 34160333

[6] McLigeyo SO. Ageing Population in Africa and Other Developing Communities: A Public Health Challenge Calling for Urgent Solutions. East Afr Med J (2002) 79(6):281–3. 10.4314/eamj.v79i6.8846 12638817

[7] Ani IJ Isiugo-Abanihe UC. Disability Among Older Adults in South-Eastern Nigeria. Int J Aging Hum Develop (2023) 97(3):374–94. 10.1177/00914150221132162 36259111

[8] Awuviry-Newton K Tavener M Wales K Byles J. Interpretative Phenomenological Analysis of the Lived Experiences of Older Adults Regarding Their Functional Activities in Ghana. J Prim Care Community Health (2020) 11:2150132720931110. 10.1177/2150132720931110 32584195 PMC7318820

[9] Groce N Kembhavi G Wirz S Lang R Trani J-F Kett M. Poverty and Disability – A Critical Review of the Literature in Low and Middle-Income Countries. Working Paper Series, (16). London: UCL Leonard Cheshire Disability and Inclusive Development Centre (2011).

[10] World Health Organisation. International Classification of Impairments, Disabilities and Handicaps: A Manual of Classification Relating to the Consequences of Diseases. Geneva: WHO (1980).

[11] Peres M Lucchetti G. Coping Strategies in Chronic Pain. Curr Pain Headache Rep (2010) 14:331–8. 10.1007/s11916-010-0137-3 20680705

[12] Leventhal H Diefenbach M Leventhal E. Illness Cognition: Using Common Sense to Understand Treatment Adherence and Affect Cognition Interactions. Cognit Ther Res (1992) 16:143–63. 10.1007/bf01173486

[13] Van Damme S Crombez G Eccleston C. Coping With Pain: A Motivational Perspective. Pain (2008) 139:1–4. 10.1016/j.pain.2008.07.022 18755548

[14] Lazarus RS Folkman S. Stress, Appraisal, and Coping. New York: Springer (1984).

[15] Carver CS Connor-Smith J. Personality and Coping. Annu Rev Psychol (2010) 61:679–704. 10.1146/annurev.psych.093008.100352 19572784

[16] Pargament KI. The Psychology of Religion and Coping: Theory, Research, Practice. New York: Guilford Press (2001).

[17] Crowther S Ironside P Spence D Smythe L. Crafting Stories in Hermeneutic Phenomenology Research: A Methodological Device. Qual Health Res (2017) 27(6):826–35. 10.1177/1049732316656161 27354387

[18] Smith J Flowers P Larkin M. Interpretative Phenomenological Analysis: Theory, Method, and Research. London: Sage (2019).

[19] Saunders B Sim J Kingstone T Baker S Waterfield J Bartlam B Saturation in Qualitative Research: Exploring its Conceptualization and Operationalization. Qual quantity (2018) 52(4):1893–907. 10.1007/s11135-017-0574-8 PMC5993836 29937585

[20] Hefferon K Gil-Rodriguez E. Interpretative Phenomenological Analysis. Psychol (2011) 24(10):756–9.

[21] Chan ZC Fung YL Chien WT. Bracketing in Phenomenology: Only Undertaken in the Data Collection and Analysis Process. Qual Rep (2013) 18:1–9. 10.46743/2160-3715/2013.1486

[22] Finlay L. Phenomenology for Therapists: Researching the Lived World. Chichester, UK: Wiley-Blackwell (2011).

[23] Tufford L Newman P. Bracketing in Qualitative Research. Qual Soc Work (2010) 11:80–96. 10.1177/1473325010368316

[24] Gearing RE. Bracketing in Research: A Typology. Qual Health Res (2004) 14:1429–52. 10.1177/1049732304270394 15538009

[25] World Health Organization. WHO STEPS Stroke Manual: The WHO STEPwise Approach to Stroke Surveillance. Geneva: World Health Organization (2006).

[26] Perry L McLaren S. Coping and Adaptation at Six Months After Stroke: Experiences With Eating Disabilities. Int J Nurs Stud (2003) 40:185–95. 10.1016/s0020-7489(02)00060-3 12559142

[27] Malone J Dadswell A The Role of Religion, Spirituality And/or Belief in Positive Ageing for Older Adults. Geriatrics (Basel, Switzerland) (2018) 3(2):28. 10.3390/geriatrics3020028 31011066 PMC6319229

[28] Shrira A Bodner E Palgi Y. The Interactive Effect of Subjective Age and Subjective Distance-To-Death on Psychological Distress of Older Adults. Aging Ment Health (2014) 18(8):1066–70. 10.1080/13607863.2014.915925 24831662

[29] Gureje O Ogunniyi A Kola L. The Profile and Impact of Probable Dementia in a Sub-Saharan African Community: Results From the Ibadan Study of Aging. J psychosomatic Res (2006) 61(3):327–33. 10.1016/j.jpsychores.2006.07.016 PMC2820716 16938510

[30] Akinyemi RO Ovbiagele B Adeniji OA Sarfo FS Abd-Allah F Adoukonou T Stroke in Africa: Profile, Progress, Prospects and Priorities. Nat Rev Neurol (2021) 17:634–56. 10.1038/s41582-021-00542-4 34526674 PMC8441961

[31] Mugisha JO Schatz EJ Randell M Kuteesa M Kowal P Negin J Chronic Disease, Risk Factors and Disability in Adults Aged 50 and Above Living With and Without HIV: Findings From the Wellbeing of Older People Study in Uganda. Glob Health Action (2016) 9:31098. 10.3402/gha.v9.31098 27225792 PMC4880619

[32] Etieyibo E Omiegbe O. Religion, Culture, and Discrimination Against Persons With Disabilities in Nigeria. Afr J Disabil (2016) 5(1):a192. 10.4102/ajod.v5i1.192 PMC5433448 28730043

[33] Abosi CO Ozoji ED. Educating the Blind: A Descriptive Approach. Ibadan: Spectrum Books (1985).

[34] Buono V Corallo F Bramanti P Marino S. Coping Strategies and Health-Related Quality of Life After Stroke. J Health Psychol (2015) 1:13. 10.1177/1359105315595117 26220458

[35] Wachholtz AB Pearce MJ Koenig H. Exploring the Relationship Between Spirituality, Coping, and Pain. J Behav Med (2007) 30:311–8. 10.1007/s10865-007-9114-7 17541817

[36] Rippentrop EA Altmaier EM Chen JJ Found EM Keffala VJ. The Relationship Between Religion/Spirituality and Physical Health, Mental Health, and Pain in a Chronic Pain Population. Pain (2005) 116:311–21. 10.1016/j.pain.2005.05.008 15979795

[37] Lazarus RS Folkman S. Transactional Theory and Research on Emotions and Coping. Eur J Personal (1987) 1(3):141–69. 10.1002/per.2410010304

[38] Caldwell JC Caldwell. P. The Cultural Context of High Fertility in Sub-Saharan Africa. Popul Develop Rev (1987) 13(3):409–37. 10.2307/1973133

[39] Togonu-Bickersteth F Akinyemi A. Ageing and National Development in Nigeria: Costly Assumptions and Challenges for the Future. Afr Popul Stud (2014) 27:361–71. 10.11564/27-2-481

[40] Kola L Owumi B. Causes of Poverty in Old Age, Not a Structural Failing? J Aging Soc Pol (2019) 31(5):467–85. 10.1080/08959420.2019.1642692 31328675

[41] Ani IJ Isiugo-Abanihe UC. Unmet Needs for Support: A Study of Older Persons With Disability in Enugu State, Nigeria. Niger J Sociol Anthropol (2022) 20(1):14–30. 10.36108/NJSA/2202.02.0110

[42] Tanyi PL Andre P Mbah P Compaore Y Senderowicz L. Care of the Elderly in Nigeria: Implications for Policy. Cogent Soc Sci (2018) 4(1):1–18. 10.1080/23311886.2018.1514688 30310826 PMC6166577

[43] Togonu-Bickersteth F Akinnawo EO Akinyele OS Ayeni E. Public Alms Solicitation Among the Yoruba Elderly in Nigeria. South Afr J Gerontol (1997) 6(2):26–31. 10.21504/sajg.v6i2.117

